# Prevalence of intestinal parasitic infections and associated risk factors among Jawi primary school children, Jawi town, north-west Ethiopia

**DOI:** 10.1186/s12879-019-3971-x

**Published:** 2019-04-25

**Authors:** Baye Sitotaw, Haileyesus Mekuriaw, Destaw Damtie

**Affiliations:** 1 Department of Biology, Bahir Dar University, Bahir Dar, Ethiopia; 2 ANRS, Jawi District Education Office, Jawi, Ethiopia

**Keywords:** Intestinal parasitic infections, Jawi town, Prevalence, Risk factor, School children

## Abstract

**Background:**

Intestinal parasitic infections (IPIs) have been major public health problems in low income countries primarily affecting school children. Previous studies in Ethiopia have shown high burden of intestinal parasitic infections in most children. In order to gain a deeper insight into the magnitude of the problem more information is needed from different localities where similar studies have not been conducted. The aim of this study was to assess the prevalence of IPIs and associated risk factors among school children in Jawi Primary School, Jawi town, north -west Ethiopia.

**Methods:**

A cross-sectional study was conducted from April to June 2017 to assess the prevalence of IPIs and associated risk factors among Jawi Primary School children, Ethiopia. A total of 422 children were selected using age-stratified systematic random sampling technique. Stool samples were examined microscopically using direct wet-mount and formal-ether concentration techniques. A structured questionnaire was used to obtain information regarding the associated risk factors. Data were analyzed using SPSS version 20 and *p* value < 0.05 was taken as statistically significant.

**Results:**

Of 406 students examined for IPIs, 235 (57.88%) were positive for one or more intestinal parasites. Single, double and triple infections were 41.9, 6.2 and 1.2%, respectively. Overall infection rate was slightly higher in males (51.85%) than in females (45.30%) though the difference was not significant. Higher prevalence rate (about 51–53%) was recorded among 6 to 18 years old children. Prevalence of *Giardia lamblia* was the highest (19.95%), followed by hookworm (13.8%), *Schistosoma mansoni* (10.3%), *Entamoeba histolytica/dispar* (5.9%), *Hymenolepsis nana* (4.2%), *Taenia species* (3%) and *Ascaris lumbricoides* (0.73%), in that order. Among the risk factors assessed, age, hand washing habit before meals, open field defecation habit, consistency of wearing shoes, habit of eating raw and unwashed vegetables, and finger nail cleanliness and trimming habit were found to be the most important predictors associated with high risk of IPIs (*p* < 0.05).

**Conclusion:**

High prevalence of IPIs among Jawi Primary school children demands improved health education on regular hand washing, latrine use, wearing shoes, cleaning finger nails, not crossing rivers with bare foot and avoiding eating raw vegetables.

**Electronic supplementary material:**

The online version of this article (10.1186/s12879-019-3971-x) contains supplementary material, which is available to authorized users.

## Background

Intestinal parasitosis refers to a group of diseases caused by one or more species of protozoa, Cestodes, trematodes and nematodes. Several infectious diseases caused by some members of these previously listed organisms have been considered as Neglected Tropical Diseases (NTDs) [1–4]. Intestinal parasitic infections (IPIs) caused by pathogenic helminthes and protozoan species are endemic throughout the world. Globally, about 3.5 billion and over 450 million people are affected and ill with parasitic infections, respectively [5]. One of the sustainable development goals of the United Nations (2030 Agenda;Goal 3.3) is to end, among others, the epidemics of NTDs through the control of the transmission of IPIs and the mitigation of possible risk factors.

Despite the improvements on the quality of medical services in terms of diagnosis of parasitic diseases, most parasitic diseases are still considered as major challenges for health centers and staff in many developing countries. And IPIs constitute to be one of the top ten major public health problems in developing countries primarily affecting school children [5]. The high prevalence rate of IPIs in developing countries depends on several factors. Socio-demographic variables associated with poverty such as reduced access to adequate sanitation, scarcity of potable water, unsafe human waste disposal systems, open field defecations and unavailability of sufficient health care as well as the prevailing bad climatic and environmental conditions are the most important risk factors.

Young children are reported to be disproportionately affected by IPIs compared to adults due to their increased nutritional requirements and a less developed immune systems. IPIs in this age group have been linked with significantly reduced growth, increased risk of protein-energy malnutrition, iron deficiency anemia and reduced cognitive/psychomotor development [1, 6, 7]. The World Health Organization (WHO) estimated that over 270 million pre-school children and over 600 million school children are living in areas where the parasites are widely transmitted and are in need of urgent treatment and preventive interventions [1]. Thus, infections by parasitic worms namely round worm (ascaris), hookworm and whipworm remain global burdens in low income countries [1, 3, 8, 9]. Global prevalence of IPIs caused by pathogenic protozoan species is also reported to be high. *E. histolytica/dispar*, the cause of amoebiasis, is one of the infective and severe diseases in 48 million individuals around the globe. In the same report, worldwide prevalence of giardiasis in both developed and developing countries was estimated to be 2.8 million new cases annually [1, 5].

In sub-Saharan Africa, the protozoan parasite (*E. histolytica* and *G. intestinalis*) and the soil transmitted helminthes (*Ascaris lumbricoides, Trichuris trichiura*, and hookworm) are the leading intestinal parasites causing significant morbidity and mortality [1, 10]. Thus, the frequency of IPIs in the region is extremely high, affecting nearly all inhabitants at some point during their lives.

The Federal Ministry of Health of Ethiopia launched the Health Extension Program in 2003 and operational since 2004 with Health Extension Workers, trained to work mainly in disease prevention and health promotion at village level. The Health Extension Workers have been serving almost all villages in rural as well as urban areas, and some improvement on health services have been shown. The program was expected to help accelerate the country’s progress in meeting some of the Millennium Development Goals.

Despite the great efforts by the ministry of health, Ethiopia is still known to be heavily affected by IPIs due to the aforementioned socio-demographic variables, behavioral factors, personal hygiene and environmental sanitation factors [1, 11–16]. Indeed, ascariasis, hookworm and trichuriasis are listed among the most common public health burdens in Ethiopia [3]. A number of other studies have also shown a considerably high prevalence of IPIs in Ethiopia. For instance, extreme prevalence (84%) was reported among Debre Elias Primary School children (north-west Ethiopia) [14] though a relatively lower overall prevalence rate of IPIs (26.53%) was reported from Mekelle town (Tigray Region, northern Ethiopia [11]. In a number of other studies conducted in the different regions of Ethiopia, overall prevalence of IPIs, ranging from 54.5 to 81%, were reported from primary school children [12, 13, 16–19].

According to clinical reports from the health center in Jawi town (north-west Ethiopia), intestinal infections are currently listed as the top reasons why people visit health facilities. However, there was no previous study conducted on the prevalence of IPIs and associated risk factors in the area. Therefore, the present study was undertaken to assess the prevalence of IPIs and associated risk factors among school children in Jawi Primary School, Jawi Town, Ethiopia.

## Methods

### Study design and study area

A school based cross-sectional parasitological study was conducted from April to June 2017 to determine the prevalence of IPIs and associated risk factors among students of Jawi Primary School, north-west Ethiopia. Jawi town is located 714 km north-west of Addis Ababa (the capital city of Ehiopia). The town is located at the geographical location of 11^0^ 33′ N and 36^0^ 29′ E at an elevation of about 1212 m above sea level. The average temperature of the town is about 38.5 °C, and average annual rainfall ranges from 1200 to 1225 mm. Based on the 2015 national housing and population censes, the total population of the town was 10,347 (5413 males and 4934 females) (Jawi District Administrative Office unpublished data, 2017).

In Jawi town, there is only one health center and two health extension offices with a total of 24 health workers: During data collection there were two public health officers who were mainly responsible for the regular activities of the clinic, but there was no medical doctor assigned for the clinic. There were twelve nurses who routinely supported the patients and facilitated the activities of the health officers. Two laboratory technicians ran the laboratory activities in the clinic based on the orders of the health officers. The rest are two pharmacists, two clinical midwives and four health extension workers. The health extension workers were dedicated to implementing the health extension packages, which are planned by the Ministry of Health and which have been launched since 2003 to improve the health development in Ethiopia. In this town, access to clean water was limited, as the result, people were forced to use various unprotected water sources.

### Study population and sample size determination

The study population was all school children enrolled in Jawi Primary School (from grades 1 to 8). The total number of enrolled students in the 2016/17 academic year was 4017 (2115 males and 1902 females). Since there was no similar study previously conducted in the area, 50% prevalence rate of IPIs was taken assuming that IPIs is significantly prevalent among students in Jawi Primary School. Accordingly, the minimum number of sample size (n) required was determined using single population proportion formula for cross-sectional surveys [20], i.e. n = Z^2^ p (1-p)/d^2^ = 1.96^2^ × 0.50 × 0.50/.05^2^ = 384 students. To compensate for the non-respondents and to minimize errors probably arising from the likelihood of non-compliance, 10% was added giving a final sample size of 422 study participants.

Study participants were selected from the students stratified into three age groups according to standards in Williams et.al [21]. The actual number of students participated in the study from each age group were selected by systematic random sampling technique using age category to include 422 school children.

### Sample collection and processing

A structured questionnaire based on known risk factors was developed in English and translated into Amharic (local language). The participants of the study (parents in the case of younger children) were interviewed to obtain socio-demographic data, behavioral and hygienic practices (see Tables 1 for details). Then, the responses were translated back into English. The questionnaire was pre-tested using thirty individuals outside the study area in a non-study sample population.

For parasitological analysis, fresh stool samples were collected. The children were instructed properly and given clean labeled collection cups along with applicator sticks, and from each student about 2 g of fresh stool was collected. At the time of collection, date of sampling, the name of the participant, age and sex was recorded for each subject on a recording format. Stool sample was preserved in 10% formalin before transported to Jawi health center laboratory. A portion of each of the stool samples was processed and examined microscopically using direct wet-mount and formal-ether concentration techniques following the procedures in WHO guidelines [22, 23]. All developmental stages of the parasites (cyst, egg, larvae and adult) were recorded.

### Limitations of the study

The study was limited to only students in Jawi Primary School. Including schools outside the town would have been better to get a bigger picture of the prevalence of IPIs in the area. The study was also limited to the presence or absence of infections without quantifying the parasite load, which may not show that the infected students were diseased.

### Data analysis

Statistical Package for Social Science (SPSS) software version 20 was used to analyze the collected data. Chi-square (χ^2^) test was performed to verify the possible association between the prevalence of IPIs and socio-demographic characteristics, behavioral factors, hygienic practices and environmental sanitation factors. Logistic regression was used to measure the strengths (the degree) of association between the prevalence of infection and the risk factors using odds ratio. In the modeling process, a univariate analysis was first done with a 0.25 level of significance to select the candidate variables for multivariate analysis. The variables, significant at the univariate analysis, were then included in the multivariate analysis [24]. Values were considered significant at *p* < 0.05.

## Results

### Socio-demographic characteristics of the study participants

From a total of 422 students selected for this study, 406 (96.2%) gave stool for intestinal parasitic examination and filled questionnaires on associated risk factors. Two hundred thirty-seven (58.37%) and 169 (41.62%) participants were from grades 1 to 4 and grades 5 to 8, respectively. The age of the participants ranged from 6 to 19 years, and was grouped into three age categories (i.e.6–11, 12–18 and 19–21). The participants who were 6 to 11 years old comprised 47% followed by the 12 to 18 age group, which accounted for 39.2% (Table 1).

**Table 1:** Socio-demographic characteristics of school children and their parents at Jawi Primary School, Jawi, Ethiopia, 2016/17

Socio-demographic variables	Categories	Frequency(n)	Percentage
Residence	Urban	237	67.24
	Rural	133	32.75
Age (year)	6–11	191	47
	12–18	159	39.2
	19–21	56	13.8
Sex	Male	216	53.2
	Female	190	46.8
Mothers’ educational status	Literate	167	41.1
	illiterate	239	58.9
Mothers occupation	Employee	43	10.6
	Merchant	82	20.2
	others	69	17
	House wife	212	52.2
Fathers educational status	Literate	275	67.7
	Illiterate	131	32.3
Fathers occupation	Employee	76	18.7
	Merchant	103	25.7
	Others	54	13.3
	Farmer	173	42.6
Family size	2	13	3.2
	3	56	13.8
	4	174	42.85
	5 and above	163	40.14
Grade level	1–4	237	58.37
	5–8	169	41.62

From the total of 406 study participants, more than half were urban dwellers; male participants were slightly greater (53.2%) than females(46.8%); more than half of students’ mothers were illiterate (could not write and read) and house wives, and close to 68% of students’ fathers were literate(and about 43% of whom were farmers). Furthermore, most of the participants (83%) came from family sizes of 4 and above (Table 1).

### Prevalence of intestinal parasitic infections among the study participants

Of the 406 students who were examined for IPIs, 235 (58%) were positive for one or more intestinal parasites. Prevalence of *Giardia lamblia* was the highest (19.95%), followed by hookworm (13.8%), *Schistosoma mansoni* (10.3%), *Entamoeba histolytica/dispar* (5.9%), *Hymenolepsis nana* (4.2%), *Taenia species* (3%) and *Ascaris lumbricoides* (0.73%), in that order (Table 2). Single, double and triple infections were identified at the rate of 170 (41.87%), 25 (6.2%) and 5 (1.2%), respectively; and statistically significant differences were observed between single and overall infections (Table 2). The prevalence of protozoa, helminthes and mixed infections were 105(25.86%), 130(32%) and 28(6.9%), respectively.

**Table 2:** Prevalence of IPIs among school children by age groups and sex at Jawi Primary School in Jawi, Ethiopia, 2016/17

Parasite species		Parasite infected student by age groups			Total	χ^2^	*P*-value
		(6–11) No.(%)	(12–18) No.(%)	(19–21) No.(%)			
Protozoa	*E.histolytica/dispar*	14(7.3)	10(6.3)	0	24(5.9)	4.250	0.119
	*G.lamblia*	36(18.8)	33(20.7)	12(21.4)	81(19.95)	0.286	0.867
Helminths	Hookworm	33(17.3)	20(12.6)	3(5.35)	56(13.8)	5.499	0.042*
	*A.lumbricoides*	1(0.52)	2(1.3)	0	3(0.73)	1.121	0.571
	*S.mansoni*	19(9.94)	19(12)	4(7.14)	42(10.34)	1.093	0.579
	*H.nana*	8(4.18)	9(5.66)	0	17(4.2)	3.307	0.191
	*Taenia sp.*	6(3.1	6(3.8)	0	12(3)	2.099	0.350
		Parasite infected student by sex					
		Male No (%)	Female No (%)	**–**	Total	χ^2^	P-value
Protozoa	*E.histolytica/dispar*	12(5.6)	12(6.3)	–	24(5.9)	0.105	0.746
	*G.lamblia*	41(19)	40(21)	–	81(19.95)	0.272	0.602
Helminths	Hookworm	36(16.7)	20(10.5)	–	56(13.8)	3.205	0.073
	*A.lumbricoides*	2(0.9)	1(0.52)	–	3(0.73)	0.220	0.639
	*S.mansoni*	26(12)	16(8.4)	–	42(10.3)	1.425	0.233
	*H.nana*	9(4.2)	8(4.2)	–	17(4.2)	0.100	0.982
	*Taenia sp.*	8(3.7)	4(2.1)	–	12(3)	0.900	0.343
Single infection	–	96(44.4)	74(38.9)	**–**	170(41.9)	3.225	0.045*
Double infection	–	13(6)	12(6.3)	–	24(6.1)	0.688	0.708
Triple infection	–	4(1.8)	1(0.52)	–	5(1.2)	0.057	0.991

* = Statistically significant (*p* < 0.05)

### Association of the different risk factors with intestinal parasitic infections

Based on chi-square test, statistically significant associations were shown between the prevalence of IPIs and the risk factors including residence, age, habit of hand washing before meals, defecation habit, habit of eating raw vegetables and unwashed fruit, consistency of wearing shoes, frequency of river water contact, and finger nail cleanliness and trimming habit(Additional file 1).

Higher prevalence rate of IPIs were observed among students coming from rural (56.4%) than urban (45%) areas. Likewise, students of 12–18 years of age (52.8%) and 6–11(50.8%) years of age were found to be more infected compared with age group between 19 and 21 years old (30.35%), and the differences in prevalence rates of IPIs between higher age groups (19–21 years old) and lower age groups (6–18 years old) were significant (*p* < 0.05).

Similarly, the prevalence of IPIs among the participants with varying habits of shoe wearing (wore always, sometimes, or did not wear at all), hand washing before meals (always or sometimes) and defecation (open field or in latrine) were statistically significant (*p* < 0.001). Moreover, the prevalence of IPIs among the participants with different habits of eating raw vegetables and unwashed fruit, river water contact (always, sometimes or not at all), and cleanliness of their finger nails (dirty and untrimmed or not) vary significantly (*p* < 0.005) Accordingly, high prevalence rates of IPIs were observed in children who did not wear shoe at all, who did not wash their hands always before meals, who had habits of open field defecation, who had frequent water contact habit (swimming, crossing river, bathing and washing clothes in rivers), who ate raw vegetables and unwashed fruits and who had dirt in their untrimmed finger nails (observed by the investigator) (Additional file 1). The rest of the factors including sex, family size, educational status and occupation of mother and father, students’ grade level, water sources, ways of disposing household wastes, latrine availability, hand washing habit after using toilet, eating raw meat, and knowledge and practice in personal hygiene and environmental sanitation were not significantly associated with the prevalence of IPIs among the school children (Additional file 1).

### Logistic regression analysis (LRA) of the most important risk factors for IPIs

The most important risk factors for IPIs among Jawi Primary School children were identified using Multivariable Logistic Regression Analyses (MLRA) (Table 3). In the modeling process, a univariate analysis was first done with a 0.25 level of significance to select the candidate variables for multivariable analysis. The variables (listed below), significant at the univariate analysis, were then included in the multivariable analysis [24]. Age, hand washing habit before meals, defecation habit, consistency of wearing shoes, habit of eating raw vegetables and unwashed fruit, and dirty and untrimmed finger nails were significantly associated with IPIs (Table 3). These six risk factors were then used for the multivariate analysis and found to be the most important predicators of IPIs among the students in Jawi Primary School.

**Table 3:** Multivariate logistic regression analysis of potential risk factors associated with IPIs among school children at Jawi Primary School in Jawi, Ethiopia, 2016/17

Risk factors	IPIs					
	Categories	Total Nn. (%)	Negative No.(%)	Positive No.(%)	AOR, 95% CI	*P*-value
Residence	Urban	273(67.2)	150(55)	123(45)	1	0.916
	Rural	133(32.75)	58(43.6)	75(56.4)	0.97(0.53,1.79)	
Age in year	6–11	191(47)	94(49.2)	97(50.8)	2.31(1.13,4.71)	0.021*
	12–18	159(39.2)	75(47.2)	84(52.8)	2.24(1.09,4.62)	
	19–21	56(13.8)	39(69.6)	17(30.4)	1	
Sex	Male	216(53.2)	104(48.1)	112(51.9)	1.29(0.82,2.03)	0.78
	female	190(46.8)	104(54.7)	86(45.3)	1	
Mothers’ occupation	Government employee	46(11.3)	20(43.5)	26(56.5)	1	0.208
	Merchant	85(20.9)	49(57.6)	36(42.4)	1.6(0.79,3.26)	
	Others	69(17)	43(62.3)	26(37.7)	1.26(0.59,2.7)	
	housewife	206(50.7)	96(46.6)	110(53.4)	2.44(1.00,5.92	
Ways of disposing household wastes	Burning	38(9.4)	25(65.8)	13(34.2)	1	
	Bury under ground	85(21.6)	46(54.1)	39(45.9)	0.52(0.24,1.12)	0.24
	On open field	283(69)	137(48.4)	146(51.9)	0.86(0.48,1.53)	
Hand washing habit before meals	Always	122(30)	72(59)	50(41)	1	0.029^*^
	Sometime	284(69.95)	136(47.9)	148(52.1)	5(1.34, 14.94)	
Defecation habit	Open field	204(50.5)	85(41.7)	119(58.3)	2.33(1.44,3.76)	< 0.001^*^
	In latrine	202(49.5)	123(60.9)	79(39.1)	1	
Eating raw vegetables and unwashed fruit	Yes	268(66)	126(47)	142(53)	1.82(1.14,2.3)	0.012*
	no	138(34)	82(59.4)	56(40.6)	1	
Source of drinking water	Protected tap water	50(12.3)	31(62)	19(38)	1	0.48
	Borehole	228(56.2)	119(52.2)	109(47.8)	0.66(0.33,1.32)	
	Unprotected spring/stream	128(31.5)	58(45.3)	70(547)	0.88(0.53,1.45)	
Having frequent water contact practices	Always	106(26.1)	53(50)	53(50)	1.72(0.97,3.07)	0.166
	Sometimes	211(52)	99(47)	112(53)	1.65(0.86,3.19)	
	Not at all	89(21.9)	56(63)	33(37)	1	
Consistency of wearing shoes	Always	111(27.3)	71(64)	40(36)	1	< 0.001*
	Sometimes	241(59.4)	121(50.2)	120(49.8)	2(1.2, 6.93)	
	Not at all	54(13.3)	16(29.6)	38(70.4)	4(1.5, 9.44)	
Dirty things in finger nails	Yes	245(60.3)	115(47)	130(53)	1.56(0.99,2.44)	0.05*
	no	161(37.43)	93(56.7)	68(43.4)	1	

Note: 1 = reference value, * = statistically significant at *p* < 0.05, AOR = adjusted odds ratio (multivariate regression model) for age, hand washing habit before meals, defecation habit, consistency of wearing shoes, habit of eating raw vegetables and unwashed fruit, and dirty finger nails

Accordingly, the likelihood of being infected by IPIs was increased two fold (AOR =2.31, CI = 1.13, 4.71, *p* = 0.021) and (AOR = 2.24 CI = 1.09, 4.62, *p* = 0.021) in both mid-childhood and early adolescence, respectively, than late adolescence; the risk of being infected by IPIs was increased by two fold in students who defecated in open field (AOR = 2.3 CI = 1.44, 3.76, *p* = 0.001) than those who used latrine; students who did not wear protective shoes were four times (AOR = 4 CI = 1.5, 9.44, *P* = 0.001) and those who sometimes wore shoes were two times more likely to be infected (AOR = 2 CI = 1.2, 6.93, *P* = 0.001) than those who always wore shoes; students who did not regularly wash hands before meals were five times (AOR = 5 CI = 1.34, 14.9 *P* = 0.029) more likely to have parasitic infections than those who washed their hands regularly before meals; participants who ate raw vegetables and unwashed fruits were two times (AOR =1.82 CI = 1.14, 2.3, *p* = 0.012) more likely to have parasitic infections than those who did not eat raw vegetables (Table 3).

### Risk factors associated with prevalence of *Giardia lamblia* and hookworm

As indicated in Table 2, *Giardia lamblia* infection was the most prevalent followed by hookworm infection. In MLRA model residence, hand washing habit before meals, defecation habit, and eating raw vegetables and unwashed fruit were predicators of *Giardia lamblia* infection. Accordingly, the risk of *G.lamblia* infection was increased by twofold in study subject who had a habit of open field defecation, consumption of raw vegetables and unwashed fruit (AOR = 2 CI = 1.1, 3.4 *P* = 0.003) and (AOR = 2.2 CI = 1.0, 4.2 *P* = 0.036) compared with those who used latrine and did not eat raw vegetables (Table 4).

**Table 4:** Multivariate logistic regression analysis of potential risk factors associated with *G.lamblia* and hookworm infection among school children at Jawi Primary School in Jawi, Ethiopia, 2016/17

Risk factors	*G. lamblia*					
	Categories	Total No. (%)	Negative No. (%)	Positive No.(%)	AOR(95%CI)	*p*-value
Residence	Urban	273(67.2)	224(94.5)	49(5.5)	1	0.96
	Rural	133(32.8)	101(76)	32(24)	0.9(0.5, 1.8)	
Hand washing habit before meals	Always	122(30)	87(71.3)	19(28.7)	1	0.01*
	Sometimes	284(69.95)	238(83.8)	62(16.2)	1.7(0.4, 3.1)	
Defecation habit	Open field	204(50.5)	152(74.5)	52(25.5)	2(1.1, 3.4)	0.003*
	In latrine	202(49.5)	173(85.6)	29(14.4)	1	
Eating raw vegetables and unwashed fruit	Yes	268(66)	211(78.7)	57(21.3)	2.2 (1.0, 4.20)	0.036*
	No	138(43)	114(82.6)	24(17.4)	1	
Hookworm						
		Total No. (%)	Negative No. (%)	positive No.(%)	AOR 95% CI	*p*-value
Age in year	6–11	191(47)	158(82.7)	33(17.3)	3.9 (1.0, 14.4)	0.037*
	12–18	159(39.2)	139(87.4)	20(12.6)	2.5 (0.6, 9.6)	
	19–21	56(13.8)	53(94.6)	3(5.4)	1	
Sex	Male	216(53.2)	180(83.3)	36(16.7)	2 (1.1, 4.3)	0.003*
	Female	190(46.8)	170(89.5)	20(10.5)	1	
Defecation habit	Open field	204(50.5)	165(80.9)	39(19.1)	3.2(1.7, 6.2)	0.001*
	In latrine	202(49.5)	185(91.6)	17(8.4)	1	
Consistency of wearing shoes	Always	111(27.3)	98(88.3)	13(11.7)	1	0.02*
	Sometimes	241(59.4)	211(87.6)	30(12.4)	2.2(1.1, 4.3)	
	Not at all	54(13.3)	41(76)	13(24)	3(1.3,7.3)	

Note: 1 = reference value, * = statistically significant at *p* < 0.05, AOR = adjusted odds ratio (multivariate regression model)for habit of hand washing before meals, defecation, eating raw vegetables and unwashed fruit for *G. lamblia* and age, sex, defecation habit and consistency of wearing shoes for hookworm infection

Age, sex, habit of open field defecation, and shoe wearing consistency were predicators of hookworm infection among Jawi Primary School children (Table 4). The male study participants were two times (AOR = 2 CI = 1.1, 4.3, P = 0.003) more at risk of having hookworm infection than females. The students between 6 and 11 years of age were four times (AOR = 3.9 CI = 1.0, 14.4, *p* = 0.037) more likely to have hookworm infection than those who were 19–21 years old. Similarly, students of 12–18 years of age were three times (AOR = 2.5 CI = 0.6, 9.6, p = 0.037) more likely to be infected than those of 19–21 years old. Study subjects who did not wear protective shoes were three times (AOR = 3 CI = 1.3, 7.3, *P* = 0.02) and those who sometimes wore shoes were two times (AOR = 2.2 CI = 1.1, 4.3, *p* = 0.02) more likely to have hookworm infection than those who always wore protective shoes.

## Discussion

Understanding the prevalence of Intestinal parasitic infections (IPIs) and associated risk factors in different localities is the key to identify high risk communities and designing appropriate intervention mechanisms. In line with this view, the present study attempted to assess the prevalence of IPIs and associated risk factors among the students at Jawi Primary School, Iawi town, north-west Ethiopia.

The overall prevalence of IPIs among the study participants was considerably high (58%). Reports from different regions of Ethiopia, too, showed elevated prevalence of IPIs ranging from 35% to as high as 80% without showing reduction [13, 16, 18, 25–30] regardless of a community-based accelerated expansion of health facilities in Ethiopia being operational since 2004. High prevalence rates of IPIs have also been reported from other developing nations including in India (49%) [31], in Nepal (51.9%) [32], in Oshoidi Logos, Nigeria (58.3%) [33], in Burkina Faso (84.7%) [34]. And even 100% prevalence rate was reported in a rural area of Peru [35] showing that IPIs are still big threats to poor society. Low socio-economic status, low family educational level, individual behavioral and personal conditions of the participants, the level of environmental sanitation, source of drinking water and low personal hygiene are widely recognized risk factors accountable for the elevated prevalence of IPIs among communities in the poor societies . In this study, sex was not found to be associated with the rate of IPIs (*p* > 0.05) (Table 3). Similar observations were reported by previous studies in Tilili (north west Ethiopia) and Babile towns (Southern Ethiopia) [29, 36, 37] though there is much evidence supporting that males are more exposed to IPIs than females [18, 30, 38, 39] mainly related to differences in gender roles. However, age and residence were found to have associations with IPIs (Table 3). High prevalence rate was recorded in young children (6–18 years old). Reports from different parts of Ethiopia [30, 38] have also shown lower rate of infection among older children. This is obviously related to their behavioral activities such as playing in contaminated soil and water, and level of awareness of transmission of IPIs. With regard to residence, those children who came from rural areas had higher IPI (56.4%) than urban dwellers (45%). This was supported by many other studies (e.g [38, 40]) and has been well documented. Poor environmental sanitation, not using toilet, inadequate water supply, low level of knowledge and practices in personal hygiene, and low living standards of the subjects are often mentioned as major factors for the high prevalence of intestinal parasites among rural dwellers than urban dwellers.

Seven types of intestinal parasites were identified from the students of Jawi Primary School (Table 2), the most prevalent being *G.lambila* (close to 20%). This high prevalence rate of *G.lamblia* infection may be attributed to one or more of the risk factors as half of the participants were found to have open field defecation habit, 66% of them ate raw vegetables and unwashed fruits, 70% had poor hand washing habit before meals and all of them lacked safe drinking water. This parasite was also found to be highly prevalent (11–23%) among other school children in Ethiopia [17–19, 40, 41]. About 6% of Jawi Primary School children were positive for another protozoan parasite, *E. histolytica/dispar,* which is considered to be low compared with previous reports from Ethiopia and some other developing nations [17–19, 28, 32, 42, 43].

Hookworm was the second most prevalent (about 14%) parasite among the study participants. It was significantly associated with defecation habit, sex, protective shoe wearing habit and age (Table 4). High prevalence of hookworm infection (11–33%) was also reported in studies at different schools in Ethiopia [17, 19, 27, 28, 37, 38]. *S. mansoni* was the third most prevalent parasite (10.3%) among the study participants*.* This is a high prevalence rate andmay most likely be related to the students’ habit of swimming in the canal water constructed near the town for sugar cane irrigation (Additional file 1). Furthermore, water contact activities such as crossing rivers, bathing in rivers, washing closes in rivers and lack of protective shoes could contribute for *S. mansoni* infection among the study participants. Reports from different parts of Ethiopia also showed high prevalence rates of *S. mansoni* infections ranging from 10 to as high as 83% [11, 12, 38, 42, 44–47]. The prevalence of *H. nana* among the study participants seems relatively low (4.2%). Of course, similar prevalence rates (4.5 to 4.7%) were reported in studies on school children in four Primary School in north-west Ethiopia [13, 19, 38] as well as a little higher rate (5.5%) in Yemen [37]. A bit higher infection rates (6.5 to 9%) were reported from some rural areas in Ethiopia as well as in other African countries [34, 48, 49]. The prevalence of *Taenia species* among the study participants was also low (3%). Similarly, low infection rates (1.5 to 3.4%) were reported from other areas in Ethiopia [13, 17] and in Nigeria [39] as well. Even much lower infection rates (0.2 to 0.8%) were reported in some regions of Ethiopia [14, 18, 44]. However, an extremely high prevalence (64%) was reported from three selected districts of west Shoa (Central Ethiopia) [50].The least frequently encountered parasite in this study was *A.lumbricoides* (0.73%) which is also found to be rare (0.6 to 1.6%) among some other school children in north west Ethiopia [14, 44]. However, much higher infection rates (8.3 to 15.5%) than observed in this study were reported from some Primary School children, still in north-west Ethiopia [17, 28, 49]. Even though the prevalence of these parasites vary from localities to localities, they remain public health burden in low income societies.

In the attempt to identify the associated risk factors for prevalence of IPI among school children in Jawi town, factors including residence, age, hand washing habit before meals, open field defecation habit, river water contact activities, eating raw vegetables and unwashed fruit, and dirty and untrimmed finger nail were significantly associated with the prevalence of IPIs (*p* < 0.05). This, more or less, concurs with previous studies conducted elsewhere in Ethiopia (e.g. [19, 28, 30]).

Multivariate logistic regression analysis was conducted to determine the degree of association between IPIs and the demographic variables as well as other risk factors (Table 3).The age of the study participants was strongly associated with IPIs, and children in both mid-childhood and early adolescence were approximately twice more at risk of IPIs than those in late adolescence. Likewise, hand washing before meals, open field defecation, shoes wearing consistency, eating raw vegetables and fingernail cleanliness were found to be predicators of IPIs among the participants of the study. Students who used to defecate in open field were at risk by twofold than those who defecated in latrine. Similarly, shoes wearing habit of students were strongly associated with IPIs. Student who did not wear protective shoes were four times likely to be infected compared with those who wore regularly, and those who wore sometimes were two times likely to be infected than those who wore always. Moreover, students who did not regularly wash hands before meals were five times likely to have IPIs than those who washed their hands regularly before meals. These observations were consistent with reports of studies elsewhere (e.g. [18, 19, 30]).

There was also strong association between the two most prevalent parasites and some risk factors. The risk of *G.lamblia* infection was increased by two fold in study subjects who had a habit of open field defecation, poor hand washing habit, and habit of eating raw vegetables and unwashed fruit compared with those who used latrine, washed their hands as well as did not eat raw vegetable (Table 4). This finding was supported by other studies [27, 28, 30, 40] suggesting that contamination of vegetables with fecal matter in farming area could be among the primary causes. Sex, age, open field defecation habit and shoes wearing consistency were found to be predicators of hookworm infection among Jawi Primary School children (Table 4). Participants who did not wear protective shoes and those wearing irregularly were threefold and twofold, respectively, more likely to be infected compared to the study subjects who always wore shoes. This result was consistent with other reports in some rural Ethiopia [28, 40].

## Conclusion

Generally, the present study showed that school children in Jawi town were heavily infected with IPIs, implying that IPIs continue to be major public health problems in low income communities. *G. lambilia*, hookworm and *S. mansoni* were the most predominant intestinal parasites detected among the school children. The most important risk factors for these infections were found to be age, inconsistency of wearing shoes, poor hand washing habit before eating, open field defecation, habit of frequent river water contact, having dirty and untrimmed finger nails, and eating uncooked vegetables. Urgent actions are needed to, at least, reduce intestinal parasitic infections through concerted approaches involving politicians (decision makers), health extension workers, school teachers, the mass media, community and religious leaders. All these bodies should design practical action plans for effective prevention and control of IPIs in the study area in general and. to create awareness among school children and their parents in particular. It is also recommended regular inspection be conducted on school children for personal hygienic practices and shoe wearing habits.

## Additional file

Additional file 1: The association of intestinal parasitic infections (IPIs) with potential risk factors among school children, Jawi Primary School, Jawi Town, Ethiopia, 2016/17. The risk factors considered for this data set were socio-demographic characteristics, behavioral factors, personal hygienic practices and factors related to environmental sanitation. (DOCX 17 kb)

## Abbreviations

AOR: Adjusted Odds Ratio
CI: Confidence interval
COR: Crude Odds Ratio
IPIs: intestinal parasitic infections
MLRA: Multivariate logistic regression analysis
SPSS: Statistical Package for Social Science
STHs: Soil Transmitted Helminthes
