# Supplementary material for: Prevalence of intestinal parasitic infections and associated risk factors among Jawi primary school children, Jawi town, north-west Ethiopia
Source: BMC Infect Dis. 2019 Apr 25;19:341. doi: 10.1186/s12879-019-3971-x (PMC6485161; doi:10.1186/s12879-019-3971-x)
Supplement: Supplementary file 1 — The association of intestinal parasitic infections (IPIs) with potential risk factors among school children, Jawi Primary School, Jawi Town, Ethiopia, 2016/17. The risk factors considered for this data set were socio-demographic characteristics, behavioral factors, personal hygienic practices and factors related to environmental sanitation. (DOCX 17 kb) [file 12879_2019_3971_MOESM1_ESM.docx]

**Additional file:** The association of IPIs with potential risk factors among school children, Jawi Primary School, Jawi Town, Ethiopia,2016/17

| **Potential risk factors** | | **Number and percentage of parasite infected student** | | | | |
| --- | --- | --- | --- | --- | --- | --- |
| Socio-demographic variables | | | | | | |
| variables | categories | Total No. (%) | Negative No. (%) | Positive No. (%) | **χ^2^** | P-value |
| Residence | Urban | 273(67.2) | 150(55) | 123(45) | 4.60 | 0.032* |
|  | Rural | 133(32.75) | 58(43.6) | 75(56.4) |  |  |
| Age in year | 6-11 | 191(47) | 94(49.2) | 97(50.8) | 9.10 | 0.011* |
|  | 12-18 | 159(39.2) | 75(47.2) | 84(52.8) |  |  |
|  | 19-21 | 56(13.8) | 39(69.6) | 17(30.4) |  |  |
| Sex | Male | 216(53.2) | 104(48.1) | 112(51.9) | 1.76 | 0.185 |
|  | female | 190(46.8) | 104(54.7) | 86(45.3) |  |  |
| Mothers’ educational status | Literate | 167(41.1) | 81(48.5) | 86(51.5) | 0.845 | 0.358 |
|  | illiterate | 239(58.9) | 127(53.1) | 112(46.9) |  |  |
| Mothers’ occupation | Government employee | 46(11.3) | 20(43.5) | 26(56.5) | 7.66 | 0.055 |
|  | Merchant | 85(20.9) | 49(57.6) | 36(42.4) |  |  |
|  | Others | 69(17) | 43(62.3) | 26(37.7) |  |  |
|  | housewife | 206(50.7) | 96(46.6) | 110(53.4) |  |  |
| Fathers’ educational status | Literate | 275(67.7) | 146(53) | 129(47) | 1.18 | 0.277 |
|  | illiterate | 131(32.3) | 62(47.3) | 69(52.7) |  |  |
| Fathers’ occupation | Government employee | 76(18.7) | 42(55.3) | 34(44.7) | 2.5 | 0.477 |
|  | Merchant | 103(25.4) | 57(55.3) | 46(44.7) |  |  |
|  | Others | 54(13.3) | 24(44.4) | 30(55.6) |  |  |
|  | farmer | 173(42.6) | 85(49.1) | 88(50.9) |  |  |
| Size of family | 2 | 13(3.2) | 8(61.5) | 5(38.5) | 1.66 | 0.88 |
|  | 3 | 56(13.8) | 21(37.5) | 35(62.5) |  |  |
|  | 4 | 174(42.85) | 87(50) | 87(50) |  |  |
|  | 5 and above | 163(40.14) | 92(56.4) | 71(43.6) |  |  |
| Grade level | 1-4 | 237(58.37) | 127(53.6) | 110(46.4) | 0.664 | 0.262 |
|  | 5-8 | 169(42.62) | 89(52.7) | 80(47.3) |  |  |
| **Behavioral and hygienic practice variables** | | | | | | |
| variables | Categories | Total No. (%) | Negative No. (%) | Positive No.(%) | χ^2^ | p-value |
| Source of drinking water | Protected tap water | 50(12.3) | 31(62) | 19(38) | 4.05 | 0.135 |
|  | Borehole | 228(56.2) | 119(52.2) | 109(47.8) |  |  |
|  | Unprotected spring/stream | 128(31.5) | 58(45.3) | 70(547) |  |  |
| Ways of disposing household wastes | Burning | 38(9.4) | 25(65.8) | 13(34.2) | 4.4 | 0.110 |
|  | Bury under ground | 85(21.6) | 46(54.1) | 39(45.9) |  |  |
|  | On open field | 283(69) | 137(48.4) | 146(51.9) |  |  |
| Hand washing habit before meals | Always | 122(30) | 72(59) | 50(41) | 4.231 | **<0.001*** |
|  | Sometimes | 284(69.95) | 136(47.9) | 148(52.1) |  |  |
| latrine availability | Present | 235(59.6) | 124(52.8) | 111(46.2) | 0.53 | 0.468 |
|  | absent | 171(40.4) | 84(49.1) | 87(50.9) |  |  |
| Hand washing habit after toilet | Always | 56(13.8) | 29(51.8) | 27(48.2) | 1.58 | 0.454 |
|  | Sometimes | 202(49.75) | 98(48.5) | 104(51.5) |  |  |
|  | No at all | 148(36.45) | 82(55) | 67(45) |  |  |
| Defecation habit | Open field | 204(50.5) | 85(41.7) | 119(58.3) | 15.01 | **<0.001*** |
|  | In latrine | 202(49.5) | 123(60.9) | 79(39.1) |  |  |
| Eating raw vegetables and unwashed fruit | Yes | 268(66) | 126(47) | 142(53) | 5.611 | **0.018*** |
|  | no | 138(34) | 82(59.4) | 56(40.6) |  |  |
| Eating raw/uncooked meat | yes | 29(7.14) | 14(48.3) | 15(51.7) | 0.214 | 0.898 |
|  | Sometimes | 255(62.8) | 128(50.2) | 127(49.8) |  |  |
|  | no | 122(30.04) | 66(54) | 56(46) |  |  |
| Frequent water contact practices swimming, crossing, bathing and washing closes in river | Always | 106(26.1) | 53(50) | 53(50) | 6.5 | **0.039*** |
|  | Sometimes | 211(52) | 99(47) | 112(53) |  |  |
|  | Not at all | 89(21.9) | 56(63) | 33(37) |  |  |
| Consistency of wearing shoes | Always | 111(27.3) | 71(64) | 40(36) | 27.4 | **<0.001*** |
|  | Sometimes | 241(59.4) | 121(50.2) | 120(49.8) |  |  |
|  | Not at all | 54(13.3) | 16(29.6) | 38(70.4) |  |  |
| Dirty things in finger nails(observed by investigator) | Yes | 245(60.3) | 115(47) | 130(53) | 4.56 | **0.033*** |
|  | no | 161(37.43) | 93(56.7) | 68(43.4) |  |  |
| knowledge and practice about personal hygiene and environmental sanitation | Good | 300(73.9) | 152(50.7) | 148(49.3) | 0.15 | 0.702 |
|  | poor | 106(26.1) | 56(52.8) | 50(47.2) |  |  |

*=Statistically significant (p<0.05)
